# Suicidal thinking, depression, and religiosity in a freedom-deprived population*

**DOI:** 10.1590/1518-8345.3713.3368

**Published:** 2020-10-19

**Authors:** Cristina Ranuzi, Tamires Gomes dos Santos, Ana Cláudia Moura Caetano Araujo, Leiner Resende Rodrigues

**Affiliations:** 1 Universidade Federal do Triangulo Mineiro, Uberaba, MG, Brazil.; 2 Scholarship holder at the Coordenação de Aperfeiçoamento de Pessoal de Nível Superior (CAPES), Brazil.

**Keywords:** Suicidal Ideation, Prisoners, Depression, Religion, Mental Health, Health Care, Ideação Suicida, Prisioneiros, Depressão, Religião, Saúde Mental, Atenção à Saúde, Ideación Suicida, Prisioneros, Depresión, Religión, Salud Mental, Atención a la Salud

## Abstract

**Objective:**

to analyze the influence of sociodemographic variables, prison context, religiosity, and symptoms of depression on the presence of suicidal thinking in a population deprived of liberty.

**Method:**

a cross-sectional study with a quantitative approach, conducted with 228 participants, based on a sociodemographic questionnaire, on the prison context, and on the presence of suicidal thinking, from the Duke Religiosity Scale and the Depression, Anxiety and Stress Scale (DASS-21).

**Results:**

the variables that showed a statistically significant correlation were the following: female gender, not having a partner, working inside the penitentiary, being a primary defendant and using controlled medication, and females are 7.2 times more likely to present suicidal thinking, for each point more in the depression score, increases by 21% in the chances and not having a partner increases the chances of thinking about suicide by three times. Although the scores of religiosity were high, they did not present a statistically significant correlation with the presence of suicidal thinking.

**Conclusion:**

the prison context is complex and contains peculiarities that cause the involvement of mental health problems, as well as self-harming thoughts. Considering the relevance of the subject at issue, this work stands out in view of the scarce scientific production on the subject.

## Abstract (pt)

**Objetivo:**

analisar a influência das variáveis sociodemográficas, de contexto prisional, religiosidade e sintomas de depressão sobre a presença do pensamento suicida em uma população privada de liberdade.

**Método:**

estudo transversal, com abordagem quantitativa, realizado com 228 participantes, a partir de um questionário sociodemográfico, de contexto prisional e presença do pensamento suicida, da escala de Religiosidade de Duke e da Escala de Depressão, Ansiedade e Estresse (DASS-21).

**Resultados:**

as variáveis que apresentaram correlação estatisticamente significativa foram: sexo feminino, não possuir companheiro(a), trabalhar dentro da penitenciária, ser réu primário e fazer uso de medicamento controlado, sendo que o sexo feminino possui 7,2 vezes mais chances de apresentar pensamento suicida, para cada ponto a mais no escore de depressão, aumenta em 21% nas chances e não possuir companheiro(a) aumenta em três vezes as chances de pensar em suicídio. Ainda que os escores de religiosidade tenham sido elevados, não apresentaram correlação estatisticamente significativa com a presença do pensamento suicida.

**Conclusão:**

o contexto prisional é complexo e contém peculiaridades que ensejam o acometimento dos agravos à saúde mental, bem como dos pensamentos autolesivos. Considerando a relevância do assunto em questão, esse trabalho se destaca diante da escassa produção científica acerca do tema.

## Abstract (es)

**Objetivo:**

analizar la influencia de variables sociodemográficas de contexto carcelario, religiosidad y síntomas de depresión en la presencia de pensamientos suicidas en una población privada de libertad.

**Método:**

estudio transversal, con enfoque cuantitativo, realizado con 228 participantes, basado en un cuestionario sociodemográfico, de contexto carcelario y la presencia de pensamientos suicidas, la Escala de Religiosidad de Duke y la Escala de Depresión, Ansiedad y Estrés (DASS-21).

**Resultados:**

las variables que mostraron una correlación estadísticamente significativa fueron: género femenino, no tener pareja, trabajar en la prisión, ser reo primario y usar medicamentos controlados, advirtiéndose que el sexo femenino presenta 7,2 veces más posibilidades de tener pensamiento suicida, por cada punto adicional en el puntaje de depresión, aumenta las posibilidades en un 21% y no tener una pareja aumenta tres veces las posibilidades de pensar en el suicidio tres Aunque los puntajes de religiosidad fueron altos, no mostraron una correlación estadísticamente significativa con la presencia de pensamientos suicidas.

**Conclusión:**

el contexto de la prisión es complejo y conlleva peculiaridades que causan el deterioro de los problemas de salud mental, así como los pensamientos autolesivos. Teniendo en cuenta la relevancia del tema en cuestión, este trabajo se destaca en vista de la escasa producción científica sobre el tema.

## Introduction

Freedom-Deprived People (FDP) constitute a risk group for suicide, presenting higher incidences when compared to the general population^(^ 1 ^-^ 2 ^)^. In the United States, for example, the occurrence of suicide among FDP is nine times higher when compared to the general population^(^ 1 ^)^. Incarceration is a traumatic experience, which implies social separation, family separation, limitation of routine activities, discrimination, poor access to the health services, permanence in a stressful environment, with precarious infrastructure and overcrowding, aspects that characterize the greater vulnerability of this population in relation to the presence of suicidal thoughts^(^ 3 ^-^ 5 ^)^.

Suicidal ideation is defined as the presence of thoughts in which the individual is the agent of his own death and the greater its magnitude and persistence, the greater the risk of eventual suicide^(^ 6 ^-^ 8 ^)^. Although the relationship between suicidal and mental disorders, especially depression and alcohol and drug abuse, are well established, factors such as conflict coping, violence, abuse or loss, isolation, and previous attempts also have a strong association with suicidal behavior^(^ 1 ^)^.

The approach to suicide is touchy, because it must be understood from the association of social, psychological, cultural, behavioral, and health factors, which act concomitantly and, because these are potentially preventable deaths, the focus should be on early identification of signs and appropriate management, and is an important target for prevention^(^ 9 ^-^ 12 ^)^. It is observed that the chances of making new attempts are ten times lower in people who received psychosocial intervention with regular care and due referral to a specialized health service^(^ 13 ^)^.

Another factor that deserves to be highlighted, regarding prevention, is religiosity, since the evidence has revealed a positive and protective relationship with mental health^(^ 14 ^-^ 16 ^)^. Participation in religious meetings attenuates the isolation experienced by the jailed population, provides psychic and spiritual well-being, creates bonds and there are indications that many of those dedicated to religious practices adopt a model of life transformation^(^ 16 ^-^ 17 ^)^.

However, few studies to date had investigated the prevalence of suicidal ideation and its correlates in a mixed sample of FDP^(^ 1 ^)^, being essential to foment new research in the area to identify risk factors as well as of protection, in order to provide instruments for professionals to support the person in the coping process^(^ 9 ^-^ 10 ^,^ 18 ^-^ 19 ^)^.

In view of the above, this study aimed to analyze the influence of sociodemographic variables, prison context, religiosity and symptoms of depression on the presence of suicidal thinking in freedom-deprived population.

## Method

A cross-sectional study, with a quantitative approach, held at the Professor Aluísio Ignácio de Oliveira state mixed penitentiary, located in the state of Minas Gerais, from May to July 2018.

The population consisted of 228 FDP, the following being considered as inclusion criteria: being male or female, convicted or provisional, and consent on participation. Participants who were unable to answer the instrument due to physical or psychological limiting factors were excluded from the study.

Sample recruitment involved non-probabilistic sampling of the study population and the sample calculation considered a prevalence of suicidal ideation of 23.7% during incarceration^(^ 12 ^)^, an accuracy of 5% and a 95% confidence interval for a finite population of 1,262 FDP. Using the Power Analysis and Sample Size application, in version 13 and introducing the values described above, a sample size of 228 individuals was obtained, and also considering a sampling loss of 20% the maximum number of interview attempts was 285.

The collection questionnaire consisted of the following instruments: sociodemographic questionnaire, questionnaire of prison context and presence of suicidal thought, Duke University Religiosity Index (Durel), and the Depression, Anxiety and Stress Scale (DASS-21).

The data referring to the sociodemographic characteristics, the prison context, and the presence of self-reported suicidal thinking, were obtained from an instrument developed by the authors and submitted to appreciation and validation by three expert judges.

The instrument addresses the following study variables for the sociodemographic characteristics: date of birth, gender, marital status, schooling level, and family income, if the participant has a religion and, if so, which one.

The characteristics of the prison context were addressed based on questions related to study and work activities, such as: if the participant studies in the prison unit and, for those who do not study, they were asked if they would like to study; if they work in the unit and, for the negative answers, they were asked if they would like to work, while for those who work, they were asked what role they play. Questions regarding the date of admission to the penitentiary, the type of detention, the type of regime, whether they are a primary or repeat offender and whether they receive visits were also included.

The following study variables were addressed, with regard to the presence of suicidal thinking: if the participant has thought about suicide at any time since imprisonment, what the frequency of that thought is, if they sought psychological assistance inside the unit, if they received psychological assistance and if they use some controlled medication.

The Durel scale was used to survey the religiosity score, a brief and easy to approach instrument, translated, and validated into Portuguese^(^ 20 ^-^ 22 ^)^. It has five items that capture three central dimensions of religiosity and that relate to health outcomes, namely: Organizational Religiosity (OR), Non-Organizational Religiosity (NOR), and Intrinsic Religiosity (IR)^(^ 20 ^,^ 22 ^)^.

OR comprises religious behaviors that occur in the context of the religious institution, such as attendance at formal religious activities. NOR, on the other hand, covers private religious behaviors, which occur without fixed locations, and can manifest themselves individually or in small family and informal groups. Finally, IR deals with a subjective dimension, which assesses how much religion can motivate or influence behaviors in the individual’s life^(^ 20 ^,^ 22 ^)^.

The first items on the scale assess OR and NOR and the last three items assess the IR. When analyzing the results, the scores of the three dimensions must be analyzed separately^(^ 21 ^)^. For both OR and NOR, the score ranges from 1 to 6 points, while IR has a variation of 3 to 15 points. It is noteworthy that the higher the total scores, the greater the religiosity^(^ 22 ^)^.

In order to proceed with the analysis, all items of the questions must be inverted to later perform the summation. For OR and NOR, the conversion results in: 1=6; 2=5; 3=4; 4=3; 3=2; 2=1 and, for IR, the conversion results in: 1=5; 2=4; 3=3; 4=2; 5=1^(^ 20 ^,^ 22 ^)^.

For the classification of the scores, low OR scores <3 and high OR scores ≥4 are considered. For NOR, <3 is low and ≥4 is high. Regarding IR, it is considered high when the scores ≥10 and low when the scores <9^(^ 23 ^)^.

The survey for the depression symptoms was carried out using the DASS-21 scale, validated and adapted to Portuguese. It is a reduced, self-administered instrument that contains 21 items that include three subscales which assess the symptoms of depression, anxiety, and stress^(^ 24 ^-^ 25 ^)^. The subscale of symptoms of depression is composed of items 3, 5, 10, 13, 16, 17 and 21, the subscale of symptoms of anxiety is composed of items 2, 4, 7, 9, 15, 19 and 20, and the subscale of symptoms of stress is composed of items 1, 6, 8, 11, 12, 14 and 18. Each item is scored from 0 to 3, with Likert-type answers, in which the individual evaluates how much each symptom was applied in the last week^(^ 25 ^)^.

The four points on the scale are the following: 0 = did not apply at all, 1 = applied to some degree or for a short time; 2 = applied to a considerable degree, or for a good part of the time, and 3 = applied a lot or most of the time. The final result is obtained by the sum of the scores of the items of each subscale, which subsequently must be multiplied by two and the classification is made according to the degree of severity^(^ 25 ^)^.

Initially, a pilot test was carried out with 10 participants, to verify the applicability of the instruments. The collection lasted approximately two months (May 22^nd^ to July 19^th^, 2018), respecting the deadline established by the Secretary of Prison Administration of the State of Minas Gerais (*Secretaria de Administração Prisional do Estado de Minas Gerais*, SEAP-MG). The interviews were conducted by the researchers, individually, with all the FDP that were handled within the prison unit, whether for routine medical care, for collecting laboratory tests, for welcoming care, for care by the Commission of Technical Classification (CTC), among others. The place for collection was a room in the health sector of the penitentiary and the duration of each interview was approximately 20 minutes.

It is noteworthy that the Free Consent Term was applied after clarification and any volunteer who presented some emotional discomfort, resulting from the application of the instruments, or presented some degree of depression, identified from the application of the scale, was welcomed by the interviewer and forwarded to psychological assistance in the prison unit, according to an agreement previously accomplished.

For statistical analysis, the Statistical Package for the Social Science (SPSS) software, version 21.0, was used. Regarding the characterization of the population according to sociodemographic variables, of the prison context and the presence or absence of suicidal thinking, categorical variables were submitted using distribution of absolute and relative frequencies and for the quantitative variables, measures of central trend and dispersion were used. In order to determine religiousness and symptoms of depression, the scale scores were summarized using centrality and dispersion measures.

As for the analysis of the relationship between sociodemographic, prison context, religiosity, and depression symptoms about the presence of suicidal thoughts, bivariate analyses were performed, such as the t test for dichotomous predictors and Pearson’s and Spearman’s correlations for quantitative and ordinal predictors, respectively. The simultaneous contribution of sociodemographic, prison context, religiosity, and depression predictors of suicidal ideation included the analysis of multiple logistic regression. Associations with p-values ≤0.05 were considered statistically significant and an alpha significance level of 5% was considered.

This study was submitted to the appreciation and approval of the SEAP-MG and to the Ethics Committee in Research with Human Beings of the Federal University of Triângulo Mineiro, under approval number 2,649,472.

## Results

The study population consisted of 228 freedom-deprived individuals, 189 (82.9%) of whom were male and 39 (17.1%), female. They were between 18 and 74 years old, with a mean of 33.48 years old, the median being 32 years old and the standard deviation being 10.2 years old. Regarding marital status, 106 (46.5%) were single.

It was verified that 98 (43%) individuals had not completed elementary school and that the most prevalent family income was one minimum wage, 65 (28.5%), followed by those who reported an income of up to three minimum wages, 58 (25.4%). The largest proportion, 210 (92.1%) reported having a religion, and of these, 104 (49.5%) were Catholics, 73 (34.8%) Evangelicals, 29 (13.8%) Spiritists, 2 (1%) Jehovah’s Witness, and 2 (1%) professed other religions.

As for the characteristics of the prison context, it was verified that 168 (73.7%) participants were convicted and that 60 (26.3%) were still awaiting trial, with 181 (79.4%) remaining in a closed regime and 47 (20 , 6%) in a semi-open regime. Most of the individuals, 141 (61.8%), reported being repeat offenders in prison. Their seclusion time varied between 0 and 5,016 days (approximately 13 years and 9 months), with a mean of 585.11 days (approximately 1 year and 6 months), a median of 329 days, and a standard deviation of 787.39 days (approximately 2 years and1 month).

Only 15 (6.6%) participants study within the prison unit and, among those who do not study, 181 (85%) say they would like to study. As for the presence of an employment relationship, only 42 (18.4%) participants had some activity; however, among the participants who did not have any activity, the majority, 172 (92.5%), demonstrated the desire to work. Among the activities offered, handicrafts were the most prevalent, 20 (47.6%). With regard to visits, 109 (47.8%) participants declared receiving them and it was identified that female participants (38.5%) receive fewer visits when compared to male (49.7%).

Table 1 displays the centrality and dispersion measures from the answers to the religiosity scale.

**Table 1:** Central tendency and dispersion measures for the dimensions of the religiosity scale applied to freedom-deprived individuals in a state mixed penitentiary. Uberaba, MG, Brazil, 2018

Dimension	Mean	Median	Standard Deviation	Minimum Value	Maximum value
OR*	4.61	5.00	1.599	1	6
NOR^†^	5.04	5.00	0.984	1	6
IR^‡^	12.07	13.00	2.662	4	15

* OR = Organizational religiosity;

† NOR = Non-organizational religiosity;

‡ IR = Intrinsic religiosity

According to the classification score, 184 (80.7%) participants had high organizational religiosity, 217 (95.2%) high non-organizational religiosity, and 187 (82%) high intrinsic religiosity.

Regarding the evaluation of the subscale of symptoms of depression, a variation from zero to 42 points was observed, with a mean of 8.50 points, median of 6.0 points, and standard deviation of 8.93 points. Table 2 contains the result of the evaluation for the subscale of depression symptoms as per severity grade.

**Table 2:** Result of the evaluation for the subscale of depression symptoms applied to freedom-deprived individuals in a state mixed penitentiary. Uberaba, MG, Brazil, 2018

Category	N	%
Normal	141	61.8
Mild	28	12.3
Moderate	38	16.7
Severe	10	4.4
Extremely severe	11	4.8

Table 3 shows the variables related to the characterization of suicidal thinking.

**Table 3:** Distribution of the frequency of the variables related to suicidal thinking in freedom-deprived individuals in a state mixed penitentiary. Uberaba, MG, Brazil, 2018

Variables	Categories	n	%
Presence of suicidal thoughts since incarceration	Yes	48	21.1
	No	180	78.9
Frequency of suicidal thinking since incarceration	One	17	35.4
	Two	8	16.7
	Three	5	10.4
	Four	3	6.3
	Five or more	15	31.3
In the presence of suicidal thought, they sought psychological assistance in the prison unit	Yes	24	50
	No	24	50
If they sought, they received psychological care	Yes	15	62.5
	No	9	37.5
Refers using controlled medication	Yes	80	35.1
	No	148	64.9

From the bivariate analysis, it is noted that the statistically significant variables were the following: being female, having no partner, working within the penitentiary, being a primary defendant, and using controlled medication, as shown in Table 4.

**Table 4:** Association between sociodemographic and prison context factors and the presence of suicidal thinking in freedom-deprived individuals in a state mixed penitentiary. Uberaba, MG, Brazil, 2018

Variables	Presence of suicidal thought				
	Yes	No	PR*	POR^†^	*p* ^‡^
Gender					
Female	20 (51.3%)	19 (48.7%)	3.462 2.188 - 5.476)	6.053 (2.873 - 12.752)	< 0.001*
Male	28 (14.8%)	161 (85.2%)			
Presence of a partner					
No	34 (28.3%)	86 (71.7%)	2.186 (1.242 - 3.848)	2.654 (1.335 - 5.280)	0.004
Yes	14 (13%)	94 (87%)			
Working inside the penitentiary					
Yes	16 (38.1%)	26 (61.9%)	2.214 (1.346 - 3.644)	2.962 (1.427 - 6.145)	0.003
No	32 (17.2%)	154 (82.8%)			
Type of defendant					
Primary	26 (29.9%)	61 (70.1%)	1.915 (1.161 - 3.161)	2.306 (1.208 - 4.400)	0.010
Repeat offender	22 (15.6%)	119 (84.4%)			
Use of controlled medication					
Yes	26 (32.5%)	54 (67.5%)	2.186 (1.328 - 3.599)	2.758 (1.438 - 5.288)	0.002
No	22 (14.9%)	126 (85.1%)			

* PR = Prevalence ratio;

† POR = Prevalence odds ratio;

‡ Chi Square Test

From the binomial multiple logistic regression, it was possible to confirm the correlations previously mentioned, as shown in Table 5. It is observed that the female gender has a 7.2 times greater chance of displaying suicidal thoughts than the male gender. As for the total depression score, it appears that for each additional point in the depression score, the chances of having suicidal thoughts increase by 21%. For the marital status variable, which in the logistic regression was dichotomized as having or not having a partner, it appears that the individuals who do not have a partner are three times more likely to think about suicide when compared to those who have a partner.

**Table 5:** Analysis of binomial multiple logistic regression having as an outcome the presence or absence of suicidal thought in freedom-deprived individuals in a state mixed penitentiary. Uberaba, MG, Brazil, 2018

Variables	*p*-value*	Adjusted POR^†^	Confidence Interval^‡^
Gender	0.001	7.197	2.358 - 21.968
Depression (total score)	<0.001	1.216	1.142 - 1.296
IR	0.477	0.941	0.796 - 1.112
Marital status	0.010	3.446	1.348 - 8.804
Type of regime	0.069	3.296	0.911 - 11.925

* p<0.005;

† POR = Prevalence odds ratio;

‡ 95% CI

## Discussion

From this study, it was found that 21.1% of the participants reported suicidal ideation since the beginning of incarceration. In the scientific literature, no study was found with a methodology similar to this, with regard to the approach of suicidal ideation among FDP, thus making it difficult to compare the data obtained with those from other authors.

The study with the methodological design closest to this was carried out in Israel, but only with 46 incarcerated women, and found a considerably higher prevalence, since more than 50% of the participants reported a history of suicidal ideation and attempted self-extermination during incarceration^(^ 26 ^)^.

Other international research studies, with different methodologies, found varied values. In Belgium, the Paykel Suicide Scale (PSS) found a prevalence of suicidal ideation of 43.1% over life, and of 23.7% during incarceration^(^ 12 ^)^. In the United States, by means of the suicide scale of the Morey Personality Inventory, it was evidenced that 16% of the participants had a clinically significant suicidal ideation in incarceration, while in Costa Rica a 10.2% prevalence was found by using the BSI scale^(^ 1 ^-^ 2 ^)^.

In Ethiopia, almost 17% of the total inmates expressed the idea of committing suicide, 16.6% have already planned it, and 11.9% have made at least one attempt since detention^(^ 27 ^)^. In Colombia, the percentages of high suicidal ideation were higher than those found in the general population. Among 154 prisoners in a prison, 14.9% had high suicidal ideation, 20.1% medium, and 64.9% low^(^ 27 ^)^.

Based on these data, it can be seen that there are still few studies on this topic, mainly those covering the FDP in Brazil^(^ 3 ^)^. Even so, it is possible to infer that the prevalence of suicidal thinking among the FDP is higher when compared to the general population^(^ 12 ^,^ 27 ^-^ 29 ^)^.

Thus, it is inferred that incarceration increases the individual’s predisposition to the involvement in suicidal ideation, due to factors such as the weakening of the social support system, overcrowding, precarious infrastructure, which require personal adaptive processes in the face of change of reality and favor the onset of symptoms such as anxiety, stress, depression, and self-injurious thoughts^(^ 3 ^,^ 27 ^-^ 28 ^,^ 30 ^-^ 33 ^)^.

As for the frequency of suicidal thinking, the majority (64.6%) of the individuals reported having thought about it more than once. As for the psychological care provided by the prison unit, half of the participants who claimed to have thought of suicide requested care at the unit and, of these, 62.5% said they had received it. For this specific information, no data were found in the literature to enable discussion. However, there is evidence that the mental health services in prison units are limited^(^ 33 ^)^.

From the bivariate analysis, it was verified that being female (p<0.001), not having a partner (p=0.004), working within the penitentiary (p=0.003), being a primary defendant (p=0.010), having self-reported depression (p<0.001) and making use of controlled medication (p=0.002) were the statistically significant variables.

From the binomial multiple logistic regression, the aforementioned correlations were confirmed, since the statistically significant variables were gender (p=0.001), total depression score (p<0.001), and marital status (p=0.010), being that female gender increases by 7.2 times the chance of having suicidal thoughts (POR=7.197), not having a partner increases it by 3 times (POR=3.466) and, for each additional point on the total score of the depression scale, they increase by 21% the chance of presenting suicidal thoughts (POR=1.216).

Reinforcing these findings, research conducted in China and the United States, the first two countries with the largest prison population in the world, indicated that psychiatric morbidities, sociodemographic variables, fragile social support, personality traits, and history of attempted suicide are significantly related to suicidal ideation in prison populations^(^ 1 ^,^ 30 ^,^ 34 ^)^.

It is noteworthy that individuals under 30 years old (p=0.04), who do not have a partner (p=0.04), with a history of intrafamily violence (p=0.03), previous suicide attempt (p=0.001), who are diagnosed with a psychiatric disorder throughout their lives (p=0.02), and incarcerated for less than one year (p=0.001) showed more probability for developing suicidal ideation^(^ 3 ^,^ 12 ^,^ 27 ^,^ 29 ^-^ 31 ^,^ 35 ^)^. Adding to this aspect, the lack of social support and being exposed to the suicidal behavior of fellow inmates increased by two times the chances of experiencing suicidal ideation^(^ 12 ^,^ 30 ^)^.

In contrast, in Europe, the decrease in the chances of suicidal ideation was significantly associated with individuals with history of previous incarceration (p=0.001), who did some work during incarceration (p=0.039), who maintained contact with family and friends (p=0.030), and with perceived safety (p=0.002)^(^ 12 ^)^.

These data are in line with a study carried out in Chile, which was based on the investigation of 132 consummated suicide occurrences in FDP, of which 66.7% had previous admissions to the prison system, 73.5% were single, 84.8% were not engaged in any work activity, and 43.2% were facing some degree of depression^(^ 3 ^)^.

As for the fact that women are more likely to have suicidal thoughts than men, the results corroborate those from other authors, who identified the same relationship both for FDP and among the general population^(^ 26 ^,^ 35 ^-^ 36 ^)^. Deaths by suicide are approximately three times higher among men than among women. Conversely, suicide attempts are, on average, three times more frequent among women^(^ 19 ^)^. The trend of men being associated with risky behaviors, higher levels of strength, easier access to more lethal means, and the fact that women are more concerned with health and seek help with less resistance, may explain this circumstance^(^ 13 ^,^ 19 ^)^.

Regarding marital status, it was verified that living alone increases the risk of suicide, with higher rates among divorced people or individuals who never married^(^ 19 ^)^. The results of this research ratify those found in the literature, since there was a significant correlation between not having a partner and the presence of suicidal ideation (p=0.004). It is added that another study found the same correlation (p=0.004) and, like other authors, states that having a partner is a protective factor^(^ 3 ^,^ 27 ^,^ 35 ^-^ 37 ^)^.

Although work during incarceration has been associated in other research studies with less suicidal ideation, this study identified an inverse correlation, since labor activity was correlated with an increase in suicidal ideation (p=0.003)^(^ 2 ^-^ 3 ^,^ 12 ^,^ 27 ^)^. Participation in work activities guarantees FDP the benefit of the remission of the penalty in the proportion of one day redeemed for every three days worked; however, its inclusion is subject to the multi-professional evaluation of the technical classification committee, which analyzes physical, psychological, behavioral, and safety conditions of the concerned individuals. It is necessary to take into account possible stress factors inherent to these activities, such as a rigorous daily review when leaving and returning from work, the reduced number of vacancies, and the consequent lack of variety of these occupations, which directs them to perform work that is not necessarily the preference or aptitude of the FDP. In addition, there is a severe demand for compliance with disciplinary rules, as any infringement of a medium or severe nature results in the suspension of this benefit^(^ 38 ^)^. It is emphasized that this condition should be better explored, in order to understand the representation of work for this population, as no article with a pertinent explanation was found.

Another variable that correlated with the existence of suicidal ideation was the fact that the FDI was a primary defendant, (p=0.010), a data consistent with the fact that a history of previous incarceration was considered a protective factor (p=0.001)^(^ 12 ^)^.

Regarding to the fact that depression has considerably increased the chances of the FDP to think about suicide, there is a consensus among specialized health groups that the mental illness usually associated with suicide is depression^(^ 39 ^)^. It is estimated that it was present in at least 50% of the consummated suicides^(^ 19 ^,^ 39 ^-^ 40 ^)^. For both the general population and the FDP, depression is an important risk factor for suicidal ideation, with a positive correlation^(^ 10 ^,^ 26 ^,^ 31 ^,^ 41 ^-^ 45 ^)^.

Regarding controlled drugs, 35.1% of the participants reported using them and a statistically significant correlation was identified with the presence of suicidal ideation; however, for this information, no studies were found that favored any comparison.

With regard to religiosity, even though the evidence indicates that it acts as a protective factor for the presence of suicidal ideation, in this study it was not possible to make such confirmation, since there was no statistically significant correlation (p=0.477/POR=0.941), in accordance with other authors^(^ 12 ^-^ 46 ^)^. There is still no evidence that religiosity significantly reduces suicide in prison, and it is suggested that perhaps this is due to the absence of studies that specifically address the impact of religiosity on the mental health of the FDP^(^ 42 ^,^ 47 ^)^.

Among the limitations of this study, it is worth highlighting the convenience sampling to the detriment of the probabilistic sampling, since the prison unit did not have the necessary number of prison officers to exclusively remove the FDP that were randomly selected. Moreover, cross-sectional studies have a unique measurement, while the follow-up of the longitudinal studies guarantees a wider data range. The fact is also added that at least one prison guard followed each participant at all times, which may have led to the underreporting of some information, incorporating possible biases.

Despite these limitations, as an important strength of this study the fact stands out that it is perhaps one of the first surveys carried out in Brazil to investigate suicidal ideation, depression, and religiosity, as well as the sociodemographic characteristics and the prison context among incarcerated men and women. Thus, the substantiality in continuing studies like this stands out, especially since it is a stigmatized and constantly growing population.

The context in which FDP are inserted is complex and has peculiarities that allow for the onset of mental health problems. Thus, better portraying the reality of this population and its profile, understanding the violence of death as a result of suicide, as well as understanding the correlated factors, can contribute to the instrumentalization of future intervention actions and to the promotion of barriers to their occurrence.

## Conclusion

This study identified that 21.1% of the participants declared suicidal ideation after incarceration and, as for the variables analyzed, being female, not having a partner, working within the penitentiary, being a primary defendant, and using controlled medications were those that had some influence on the presence of this thought. It was also verified that being female increases by 7.2 times the chance of having suicidal thoughts, that not having a partner increases it by 3 times and that, for each additional point on the total score of the depression scale, the increase is 21% in the chances of presenting the same thought. It is concluded that the proposed objectives were achieved and that the study contributes to measures that can help in the identification of people at risk, such as the elaboration of new intervention proposals, the prevention of suicidal behavior, and health promotion.
